# Notes on Materia Medica and Pharmacy

**Published:** 1885-03

**Authors:** 


*Notes on Materia Medica and Pharmacy.* By FREDERICK T. ROBERTS, M.D., B.Sc., F.R.C.P. London: H. K. LEWIS, 136 Gower Street. 1884.

In reviewing recently Dr. Mitchell Bruce's *Manual of Materia Medica*, we called attention to the fact that admirable as his book was, as a whole, it appeared to treat somewhat insufficiently the departments of Pharmacy and Pharmaceutical Chemistry, so that the student preparing for an examination in Materia Medica would find it necessary to seek for further information elsewhere on these subjects. Dr. F. T. Roberts has now issued that part of the notes for his course of lectures on Materia Medica at University College, which relates to Materia Medica, Pharmacy and Pharmaceutical Chemistry. This excellent manual will form the complement of Dr. M. Bruce's book, and if conciseness has been aimed at until something like aridity has been obtained, the book will be but so much the better suited for the student, whose knowledge of Materia Medica for examination purposes is usually limited to the acquirement of a few fugitive statistics and facts. To assist the student in this process we think Dr. Roberts' book will be of very high value, the organic materia medica and the summary of officinal preparations being arranged in a most convenient form. Dr. Roberts, having thus provided his students with a manual of pharmaceutical mnemonics, intends to lecture in future on Therapeutics only. *O fortunatus nimium!*

